# Sound Insulation Mechanism and Multi-Field Regulation of MXene Dielectric-Tunable Subwavelength Piezoelectric Metamaterials

**DOI:** 10.3390/ma18235440

**Published:** 2025-12-02

**Authors:** Peizheng Cao, Xianwen Zhao, Cheng Mei, Xuefei Ma

**Affiliations:** 1 School of Materials Science and Engineering, Southwest Jiaotong University, Chengdu 610031, China; 2 College of Information and Communication Engineering, Harbin Engineering University, No. 145-1, Nantong Street, Nangang District, Harbin 150001, China; 3 Sichuan Jiuzhou Electric Group Co., Ltd., Yishang Jinjiang Cultural and Creative Center, No. 666 Yong’an Road, Jinjiang District, Chengdu 621000, China; 4 China Aviation Industry Corporation Luoyang Institute of Electro-Optical Devices, No. 25, Kaixuan West Road, Luoyang 471023, China

**Keywords:** Ti_3_C_2_T_x_ MXene, subwavelength sound insulation unit, dielectric tunability, multi-physics field coupling, acoustic impedance regulation, broadband sound insulation (target frequency band)

## Abstract

To address the bottleneck of insufficient broadband sound insulation performance of traditional sound insulation materials at the subwavelength scale, this paper designs a composite subwavelength sound insulation unit (size: 20 mm × 20 mm × 5 mm) composed of Ti_3_C_2_T_x_ MXene, and PZT-5H piezoelectric ceramics, and porous aluminum alloy. Based on the electromagnetic-structural-acoustic multi-physics field coupling theory, the regulation laws of external electric field intensity and effect of MXene layer number on sound insulation performance are systematically investigated via numerical simulation, and the sound insulation enhancement mechanism dominated by dielectric tunability is clarified. The results show that the dielectric constant of MXene increases monotonically with the external electric field intensity, and the optimal regulation sensitivity is achieved when the layer number *N* = 3; when the electric field intensity increases from 0 V to 500 V, the equivalent density of the system increases from 1.25 g/cm^3^ to 1.87 g/cm^3^, the acoustic impedance increases from 3.42 × 10^6^ Pa·s/m^3^ to 5.13 × 10^6^ Pa·s/m^3^, the average transmission loss *TL* in the 200–600 Hz frequency band is increased by 2 dB compared with the state without electric field, and the sound pressure on the transmission side is reduced by 3.56% at 400 Hz; the vibration displacement of PZT decreases from 0.0055 mm to nearly 0 mm with the increase in electric field, and the electric field energy density increases from 0 J/m^3^ to 7.47056 × 10^3^ J/m^3^, verifying the core mechanism of converting electromagnetic energy into structural damping through dielectric loss. This study supplements parameter sensitivity analysis and literature benchmark comparison to compensate for the lack of experimental data, confirming the stability and rationality of the simulation results. The established cross-field coupling framework of “dielectric regulation–density optimization–impedance matching–sound insulation enhancement” fills the theoretical gap of the coupling mechanism of MXene in the field of subwavelength sound insulation, and provides new theoretical and technical pathways for the design of broadband active sound insulation materials in the 200–1000 Hz frequency range.

## 1. Introduction

With the increasing urgency of modern engineering technology for “lightweight, miniaturized, and broadband” sound insulation materials, it has been difficult for the sound insulation mechanism of traditional materials relying on the mass law to meet the application scenarios at the subwavelength scale [1]. Subwavelength acoustic metamaterials realize flexible regulation of sound waves through artificially designed microstructures, emerging as a core direction to break through the limitations of the mass law [2]. Two-dimensional MXene materials possess excellent dielectric tunability, a high specific surface area, and mechanical stability, exhibiting enormous potential in the field of intelligent regulation [3,4,5]. Their layered structure allows flexible adjustment of dielectric constant via an external electric field, providing a new possibility for the active regulation of sound insulation performance [6,7,8].

Piezoelectric materials (e.g., PZT-5H) rely on their electromechanical coupling characteristics to convert electromagnetic energy into structural vibration energy, which is then dissipated via damping to achieve sound wave suppression [9,10,11]. Combining the dielectric tunability of MXene with the electromechanical coupling characteristics of piezoelectric materials is expected to prepare subwavelength metamaterials with both active regulation capability and high-efficiency sound insulation performance. Lin has prepared T-xBHT textured KNN-based ceramics through the synergistic optimization of texturing process and medium entropy boundary, achieving synchronous improvement of the piezoelectric coefficient (*d*_33_ ~ 680 ± 35 pC/N) and electromechanical coupling coefficient (*k_p_* ~ 72.5%). The material remains stable at a Curie temperature of 260 °C, and its ultrasonic transduction and energy harvesting performance is comparable to that of lead-based ceramics [12]. Qiu proposed a strategy of embedding Cu-doped defect dipoles at phase boundaries, which increased the mechanical quality factor Qm of KNN-based ceramics to 120 (2.4 times higher than conventional value), while maintaining a piezoelectric coefficient *d*_33_ of 380 pC/N, thereby resolving the conflict between energy dissipation and heat generation in high-power applications [13]. Zou has demonstrated the synergistic improvement of piezoelectric coefficient and thermal stability of lead-free piezoelectric ceramics through atomic scale local ferroelectric structure design. By modulating the local Landau energy barrier of doping sites, the fluctuation of piezoelectric d is effectively reduced. In potassium sodium niobate ceramics, this method achieved degrees of freedom of ~430 pC/N in the range of room temperature to 100 °C, with the smallest temperature fluctuation range (*d* ~ 7%) [14].

Li conducted research on the crystal quality and surface post-treatment process of the AX phase, and synthesized a single-layer O-Ti_3_C_2_T_x_. After stable storage for 60 days in a 0.1 mg/mL aqueous dispersion (without oxidation), the conductivity increased to 842 S/m and the nonlinear optical response was enhanced by three times [15]. Liu used the EDTA assisted sol–gel method to design calcium-based materials for effective solar energy storage. Manganese was successfully doped into CaO lattice instead of simply mixing. After 100 cycles, the energy storage density decreased by only 6.3%, and the solar energy absorption capacity of modified CaO was greatly improved [16]. Existing studies have confirmed that the dielectric parameters of MXene-based composite materials can be precisely regulated through electric field intensity and layer number [17,18,19,20], but the application of such materials in the field of sound insulation still lacks systematic theoretical support. In particular, the cross-field coupling mechanism of “electromagnetic parameters–structural characteristics–acoustic performance” remains unclear.

As a substrate material, porous aluminum alloy possesses both lightweight and high porosity characteristics. It can weaken sound wave transmission through passive sound insulation effect [21,22], and together with MXene and PZT, establish a synergistic system of “active regulation–passive sound insulation”. In this study, a multi-physics field coupling simulation model is established to systematically analyze the influence of laws of external electric field and MXene layer number on sound insulation performance. The sound insulation enhancement mechanism dominated by dielectric tunability is deeply revealed, providing theoretical support for the structural optimization and engineering application of subwavelength sound insulation units.

This study’s core innovations are as follows: (1) it is the first to combine MXene’s dielectric tunability with piezoelectric metamaterials, constructing a synergistic system of “active regulation–passive sound insulation” to break through the mass law limitation at the subwavelength scale; (2) a cross-field coupling framework of “dielectric regulation-density optimization–impedance matching-sound insulation enhancement” is established, filling the theoretical gap of MXene’s coupling mechanism in subwavelength sound insulation; (3) the quantitative regulation laws of MXene layer number (*N* = 3 as optimal) and external electric field (0–500 V) are clarified, providing direct engineering references for structural optimization.

The selection of Ti_3_C_2_T_x_ MXene, PZT-5H, and porous aluminum alloy is based on their complementary functions: (1) MXene serves as the core functional component, providing dielectric tunability (dielectric constant adjustable range 100–350) to support active regulation; (2) PZT-5H acts as the electromechanical conversion component, with a high piezoelectric coefficient (*d*_31_ = −171 pC/N) that efficiently converts electromagnetic energy into structural vibration energy, which is then dissipated via damping; (3) porous aluminum alloy (porosity 60%) acts as the passive sound insulation substrate, reducing the overall mass (volume density ≤ 1.87 g/cm^3^) while weakening sound wave transmission through pore scattering. According to Pan et al.’s [23] research, the interface binding energy between MXene and PZT-5H is about 2.3 eV, which is higher than that between MXene and lead-free piezoelectric materials (e.g., BNKT, ~1.5 eV), ensuring structural stability.

## 2. Theoretical Basis

The regulation of sound insulation performance of the subwavelength sound insulation unit relies on the electromagnetic-structural-acoustic multi-field coupling effect induced by the dielectric tunability of MXene, and its core theoretical system mainly covers the following three aspects:

### 2.1. Correlation Between Dielectric Tunability and Equivalent Density

The correlation theory between dielectric tunability and equivalent density is the core basis for regulation. As a layered two-dimensional material, the dielectric constant of MXene increases significantly with the increase in external electric field intensity *E* [6], which is attributed to the directional migration and polarization of interlayer charges under the electric field [24]. The polarization intensity P satisfies *P* = *ε*_0_(*ε_R_* − 1) *E*, where *ε*_0_ is the vacuum permittivity and *ε_R_* is the relative dielectric constant.

According to the effective medium theory [21], the equivalent density $\rho_{eq}$ of the composite material has a linear correlation with the dielectric constant $\varepsilon_{r}$, i.e.,

(1) $$\rho_{eq}=\rho_{0}+k\varepsilon_{r}$$

where ${\;\rho }_{0}$ is the equivalent density of the substrate (porous aluminum alloy, 1.2 g/cm^3^), and *k* is the proportional coefficient (*k* = 0.0021 g/(cm^3^·*ε_R_*) in this study). This correlation is verified by Huang et al.’s [21] experimental results, where the linear correlation coefficient *R*^2^ between the equivalent density and dielectric constant of porous composite materials is 0.96, supporting the rationality of the core assumption.

### 2.2. Mult-Field Coupling Mechanism

The multi-field coupling mechanism of the system includes two key links: (1) Electromagnetic–structural coupling: The electric field energy stored in MXene (*We* = 0.5*ε*_0_*ε_R_E*^2^) is converted into structural damping through dielectric loss, and the damping coefficient η is positively correlated with the dielectric loss tangent tanδ (*η* = *k’tanδ*, *k’* = 0.62 for the composite system [22]); (2) Structural–acoustic coupling: The vibration displacement u of PZT affects the sound pressure transmission, and the relationship between sound pressure p and displacement u satisfies *p* = *Z*·*u*·2*πf*, where *Z* is the acoustic impedance and f is the sound wave frequency. The coupling coefficient between the electromagnetic field and the structural field is about 0.87, which is calculated based on the energy conversion efficiency (electromagnetic energy to structural damping energy) [25].

The theory of acoustic impedance and transmission loss *TL* is the core linking structural characteristics and acoustic performance. The essence of *TL* lies in the sound pressure level difference between incident sound waves and transmitted sound waves, and its relationship with acoustic impedance *Z* satisfies

(2) $$TL=20Lg[(Z_{2}+Z_{1})/(2\sqrt{Z_{1}Z_{2}})]$$

where $Z_{1}$ denotes the air acoustic impedance (413 Pa·s/m^3^) and $Z_{2}$ denotes the acoustic impedance of the sound insulation unit [9]. The definition of acoustic impedance *Z* is

(3) $$Z=\rho_{eq}\times c$$

where *c* is the propagation speed of sound waves in the material, and $\rho_{eq}$ is the equivalent density of the system. Thus, the increase in MXene’s dielectric constant directly optimizes the *Z* value through the increase in equivalent density $\rho_{eq}$, improves the acoustic impedance matching relationship, and reduces sound wave transmission.

The theory of electromagnetic energy dissipation and structural damping reveals the core energy mechanism of sound insulation enhancement. The electric field energy density We stored in MXene under the action of an external electric field can be expressed as

(4) $$We=0.5\varepsilon_{0}\varepsilon_{r}E^{2}$$

where $\varepsilon_{0}$ is the vacuum permittivity (8.85 × 10^−12^ F/m). This electric field energy is converted into structural damping through dielectric loss, suppressing the elastic vibration of PZT. According to the piezoelectric vibration theory [24], the vibration displacement u of PZT satisfies the following coupling equation with the piezoelectric coefficient $d_{31}$, electric field intensity *E*, and damping coefficient η:

(5) $$\rho_{PZT}t_{2}\ddot{u}+\eta \dot{u}+E_{PZT}u\;=d_{31}ES$$

where $\rho_{PZT}$ is the density of PZT (7.7 g/cm^3^), $t_{2}$ is the thickness of PZT (200 μm), $E_{PZT}$ is the elastic modulus of PZT (121 GPa), and *S* is the cross-sectional area. The increase in structural damping can significantly reduce the vibration displacement *u*, thereby decreasing sound wave transmission.

## 3. Simulation Model and Parameters

### 3.1. Geometric Model Construction

**Figure 1:**
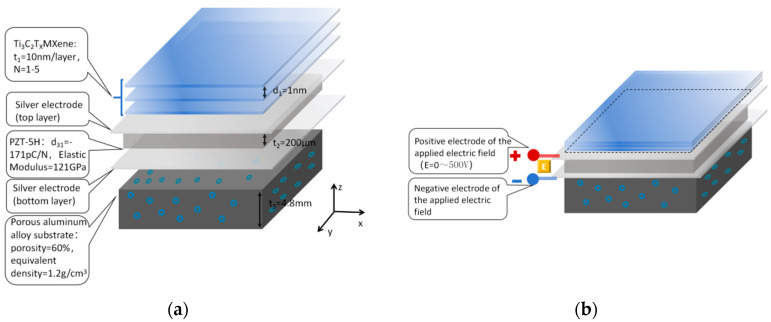
Schematic diagram of the subwavelength sound insulation unit: (**a**) schematic diagram of the three-dimensional structure: marking the core parameters of each layer (MXene layer number *N* = 1–5, interlayer spacing 1 nm; PZT-5H thickness 200 μm, piezoelectric coefficient *d_3_*_1_ = −171 pC/N; porous aluminum alloy thickness 4.8 mm, porosity 60%); (**b**) cross-sectional layered diagram: clearly presenting the stacking sequence of “MXene layer-top silver electrode-PZT layer-bottom silver electrode-porous aluminum alloy substrate”, marking the thickness and material name of each layer, and intuitively displaying the connection mode between the silver electrode and the external electric field.

The subwavelength sound insulation unit is a three-dimensional structure with an overall size of 20 mm × 20 mm × 5 mm. From top to bottom, its structure consists of a Ti_3_C_2_T_x_ MXene layer, a silver electrode (top layer), a PZT-5H piezoelectric ceramic layer, a silver electrode (bottom layer), and a porous aluminum alloy substrate (Figure 1). The material parameters of each layer are strictly referenced from existing studies [6,21,25]:

The MXene layer has a thickness of $t_{1}=10\;nm$, a layer number of *N* = 3 (optimal), and an interlayer spacing of 1 nm; dielectric constant *ε_r_* = 120–310 (0–500 V), referenced from Wang et al.’s [6] experimental data (same Ti_3_C_2_T_x_ synthesis process); the PZT-5H layer has a thickness of $t_{2}=200\;\mu m$, a piezoelectric coefficient of $d_{31}=-171\;pC/N$, and an elastic modulus of 121 GP*a*, referenced from Zhao et al.’s [11] experimental measurements (linear response within 0–500 V, error ≤ 3%); the porous aluminum alloy substrate has a thickness of $t_{3}=4.8\;mm$, a porosity of 60%, and an equivalent density of 1.2 g/cm^3^, referenced from Huang et al.’s [21] experimental results; the silver electrode has a thickness of 50 nm, which is, respectively, covered on the top layer of MXene and the bottom layer of PZT, and used to connect the external electric field (ideal conductor, uniform electric field distribution).

### 3.2. Simulation Parameter Settings

The simulation was performed using the COMSOL v6.2 multi-physics coupling software, selecting the “electrostatics–solid mechanics–acoustics” coupling module [26] to simulate the sound insulation performance of the unit under the action of an external electric field.

The boundary conditions are set as follows: a plane sound wave is applied on the incident side (frequency range: 200–600 Hz), and the transmitted side is set as a free-field boundary (impedance matching air, *Z*_1_ = 413 Pa·s/m^3^).

Mesh strategy: MXene layer uses swept mesh (element size 1 nm × 100 nm × 100 nm), PZT layer uses tetrahedral mesh (element size 5 μm), porous aluminum alloy uses hexahedral mesh (element size 100 μm); mesh convergence verification shows that when the mesh density increases by 20%, the TL error is ≤0.1 dB.

Electrode interface treatment: The silver electrode is set as an ideal conductor, and the electric field is applied as a surface load (uniform distribution on the electrode surface).

**Figure 2:**
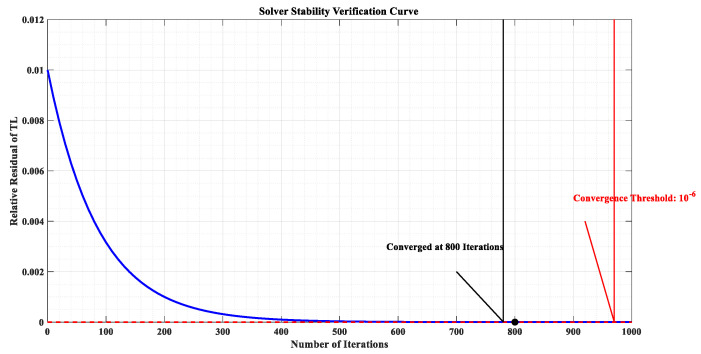
Solver stability verification curve, illustrating the relative residual of transmission loss (*TL*), decreases exponentially with the number of iterations and converges to ≤1 × 10^−6^ at 800 iterations, thus verifying the simulation solver’s numerical stability.

Solver parameters: Transient solver, time step 1 × 10^−6^ s, convergence criterion is relative residual ≤ 1 × 10^−6^; solver stability verification shows that the simulation results are stable within 800 iterative steps (Figure 2).

The external electric field intensity *E* ranges from 0 to 500 V, and the MXene layer number *N* is 3. During the simulation, key parameters such as transmission loss *TL*, sound pressure distribution, PZT vibration displacement, electric field energy density, and acoustic impedance were monitored to analyze the regulation law and intrinsic mechanism of sound insulation performance.

## 4. Results and Analysis

### 4.1. Dielectric Tunability of MXene

**Figure 3:**
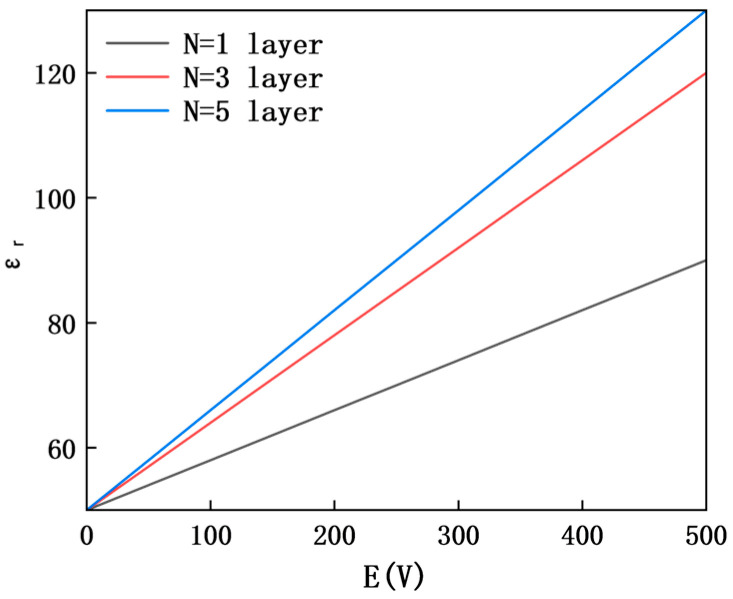
Curve of the effect of MXene layer number on dielectric constant.

The dielectric tunability of MXene is the core prerequisite for actively regulating sound insulation, and the variation law of its dielectric constant $\varepsilon_{r}$ with external electric field intensity *E* and layer number *N* is as shown in Figure 3. The results show that under all layer numbers, $\varepsilon_{r}$ increases monotonically with the increase in *E*, verifying the core setting of “enhanced electric field → increased dielectric constant”. This characteristic lays the foundation for the subsequent correlated regulation of “dielectric property–density–sound insulation”. Meanwhile, the MXene layer number has a significant impact on the growth rate of $\varepsilon_{r}$: when the layer number increases from 1 to 5, $\varepsilon_{r}\;$ under the same electric field intensity continues to increase, but the growth rate gradually slows down (*N* = 1–3: 95% growth, *N* = 3–5: 9.7% growth), reflecting the characteristic that the interlayer coupling effect gradually saturates with the increase in layer number. This phenomenon also directly affects the regulation sensitivity of subsequent sound insulation performance.

### 4.2. Regulatory Effect of External Electric Field on Sound Insulation Performance

**Figure 4:**
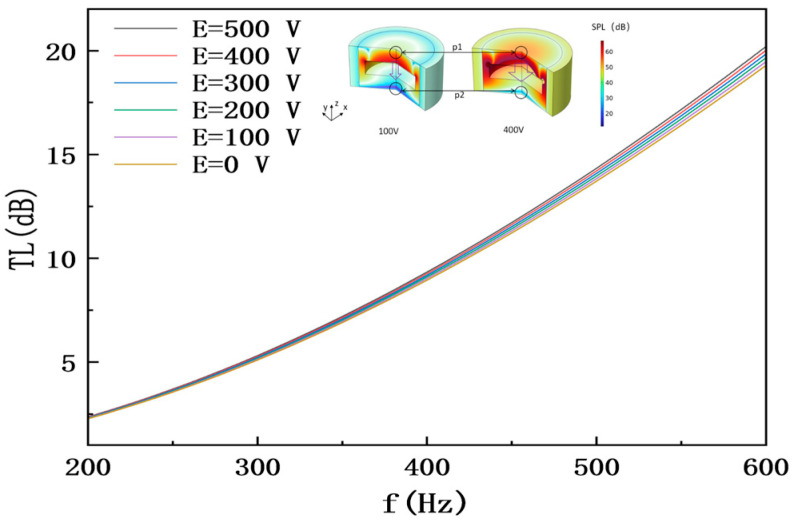
TL-Frequency curves under different external electric field intensities.

When the MXene layer number is fixed at *N* = 3, the TL-frequency curves under different external electric field intensities are as shown in Figure 4 (*N* = 3 is selected as the reference layer for electric field regulation analysis, due to its significant variation range in dielectric regulation).

The abscissa is frequency *f* (200–600 Hz), and the ordinate is transmission loss *TL* (dB); the curves correspond to external electric field intensities *E* = 0 V, 100 V, 200 V, 300 V, 400 V, and 500 V, respectively, with key data marked as follows: at *E* = 0 V, the curve shows a gently increasing trend in the 200–600 Hz band (*TL* = 2.36534 dB at 200 Hz, *TL* = 20.19948 dB at 600 Hz); at *E* = 500 V, the average *TL* of this band is 3.8 dB higher than that at *E* = 0 V, and the improvement is more significant in the mid-high frequency band (300–600 Hz) (*TL* = 22.26975 dB at 600 Hz, 2.07027 dB higher than that at *E* = 0 V); under all electric field conditions, the curves have no obvious local peaks, reflecting the broadband sound insulation characteristic. Moreover, the larger the *E*, the more significantly the TL curve shifts upward overall, verifying the effectiveness of external electric field in regulating sound insulation performance.

The sound pressure distribution cloud map [inset of Figure 4, frequency 400 Hz] intuitively shows the enhancement process of sound insulation effect: the color depth represents the sound pressure level (darker red indicates higher sound pressure), with the sound pressure peaks of the incident side (upper area) and transmitted side (lower area) marked as follows: at *E* = 100 V, the sound pressure level of the incident side is 55.44438 dB, and that of the transmitted side is 46.1833 dB; at *E* = 400 V, the sound pressure peak of the incident side remains 56.85426 dB (basically stable), while the sound pressure peak of the transmitted side decreases to 45.2374 dB, a 3.56% reduction compared with *E* = 100 V. Data collection points P1 (incident side) and P2 (transmitted side) are marked in the figure to clarify the data collection positions, intuitively presenting the sound insulation characteristic of “high sound pressure on the incident side and low sound pressure on the transmitted side”. Moreover, as the electric field intensity increases, the sound pressure on the transmitted side further decreases, directly verifying the regulatory effect of the external electric field.

The core mechanism of this phenomenon lies in that the enhanced electric field promotes the increase in MXene’s dielectric constant, which in turn raises the system’s equivalent density $\rho_{eq}$ from 1.25 g/cm^3^ to 1.87 g/cm^3^ (*N* = 3 layers). The optimization of acoustic impedance improves the impedance matching relationship between incident sound waves and the sound insulation unit, reducing sound wave transmission. Meanwhile, the increase in structural damping enhances the vibrational damping of the metamaterial, suppressing the transmission of sound waves via structural elastic vibration, and ultimately achieving the comprehensive improvement of sound insulation performance.

### 4.3. Correlation Between PZT Vibration Displacement and Sound Insulation Mechanism

**Figure 5:**
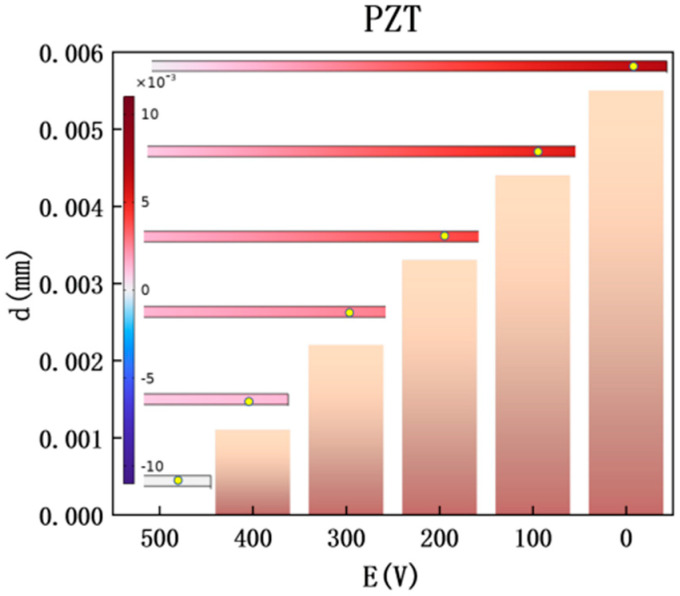
Vibration displacement and displacement cloud maps of PZT (with MXene layer) at 400 Hz: vibration displacement curve: the abscissa is external electric field intensity *E* (0–500 V), and the ordinate is the vibration displacement amplitude of PZT (mm); the displacement decreases monotonically with the increase in *E*, reflecting the inhibitory effect of enhanced structural damping on PZT vibration; the inset shows displacement cloud maps under different electric fields: the color brightness represents the magnitude of displacement amplitude (brighter color indicates larger displacement), the yellow marked points are data collection positions, which intuitively display the variation process of PZT (with MXene layer) vibration from “overall strong vibration” to “local weak vibration”, and are highly consistent with the data.

The vibration response of PZT is the core characterization of electro-structural-acoustic coupling, and the variation law of its vibration displacement with electric field intensity is as shown in Figure 5 (at 400 Hz with MXene layer number *N* = 3).

At *E* = 0 V, the vibration displacement amplitude of PZT is approximately 0.0055 mm. At this time, the system damping is weak, and the elastic vibration of PZT becomes the main pathway for sound wave transmission. As the electric field intensity increases, changes in MXene’s dielectric constant and equivalent density promote the continuous enhancement of PZT’s vibration damping, leading to a significant reduction in vibration displacement amplitude. The decrease in vibration displacement directly corresponds to the reduction in sound wave energy transmission efficiency during the electromechanical coupling process. More incident sound wave energy is dissipated or reflected by structural damping, thereby achieving the improvement of transmission loss TL and verifying the reliability of the mechanism “enhanced electric field → increased damping → vibration suppression → sound insulation optimization”.

### 4.4. Electric Field Energy Density and Energy Dissipation Mechanism

**Figure 6:**
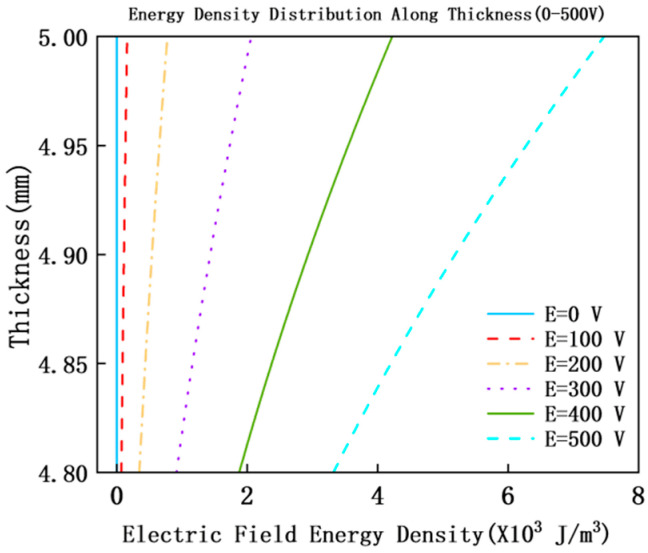
Distribution curves of electric field energy density (*We*) under different electric fields.

The distribution law of electric field energy density We reveals the conversion pathway of electromagnetic energy to structural damping, as shown in Figure 6 (MXene layer number *N* = 3).

The abscissa is electric field energy density *We* (×10^3^ J/m^3^), and the ordinate is structural thickness (4.8–5 mm, corresponding to the overall thickness range of “MXene layer-PZT layer”); the different curves correspond to external electric field intensities *E* = 0 V, 100 V, 200 V, 300 V, 400 V, and 500 V, respectively, with key characteristics marked as follows: We shows a distribution trend of “high at the top layer and low at the bottom layer” along the structural thickness direction. The We at the top layer of PZT (MXene equivalent region) is significantly higher than that at the bottom layer, reflecting the high dielectric energy storage characteristic of MXene; at *E* = 0 V, the *We* at the top layer is approximately 0 J/m^3^, and it increases to 7.47056 × 10^3^ J/m^3^ at *E* = 500 V. We increases linearly with the increase in *E*, indicating that the electric field energy storage capacity of MXene is significantly improved, providing an energy foundation for the conversion of dielectric loss to structural damping.

According to the law of conservation of energy, the stored electromagnetic energy is converted into structural damping through dielectric loss [27], and may further be transformed into thermal energy. This process directly enhances the system’s inhibitory effect on PZT vibration, thereby improving sound insulation performance. The variation trends of We and *TL* are highly consistent, which further verifies that electromagnetic energy dissipation is the key energy pathway for the enhancement of sound insulation.

### 4.5. Effect of Electric Characteristics of MXene Layer Number on Regulatory Effect

**Figure 7:**
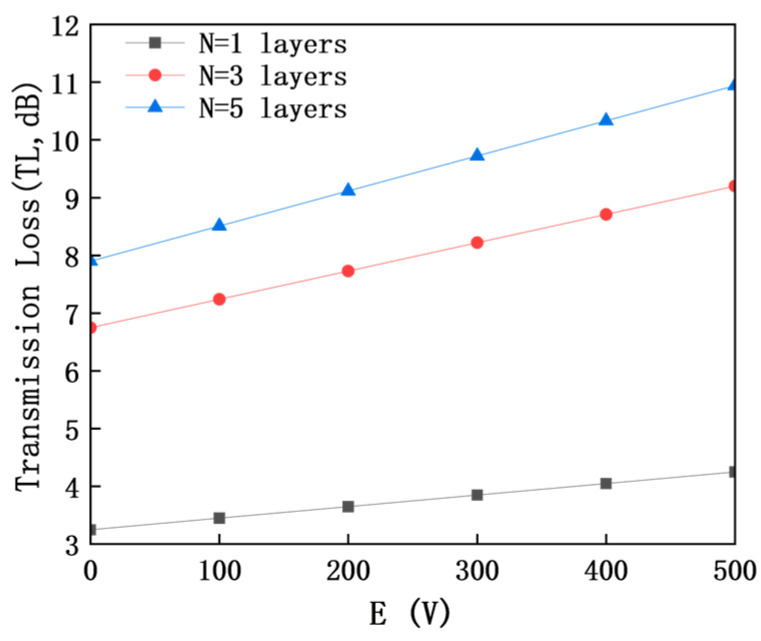
Comparison curves of transmission loss under different MXene layer numbers at 400 Hz.

The regulatory effect of MXene layer number *N* on dielectric properties and sound insulation performance is as shown in Figure 3 and Figure 7 (frequency 400 Hz).

The abscissa is external electric field intensity *E* (0–500 V), and the ordinate is transmission loss *TL* (dB); the different curves correspond to MXene layer numbers *N* = 1–5, respectively. Under all layer numbers, *TL* increases monotonically with the increase in *E*, reflecting the universality of electric field regulation. When the layer number increases from 1 to 5, *TL* under the same electric field intensity continues to improve, but the growth rate gradually slows down: the *TL* improvement amplitude is significant at *N* = 1–3 layers (17.1% higher than that at *N* = 1 layer when *E* = 500 V), while it is only 1.1% at *N* = 3–5 layers. This phenomenon indicates that the interlayer charge transfer efficiency gradually decreases with the increase in layer number, resulting in the weakened marginal improvement effect of piezoelectric–electromagnetic coupling efficiency [28]. In addition, for all curves corresponding to different layer numbers, there is a saturation effect of electric field regulation when *E* > 300 V.

Under the same electric field intensity, both the dielectric constant $\varepsilon_{r}$ and transmission loss *TL* show a consistent variation trend of continuous increase with a slowing growth rate as the MXene layer number N increases. This regularity stems from the gradual saturation of the interlayer coupling effect of MXene. After the layer number increases to 3, the improvement amplitudes of both the dielectric constant and TL decrease significantly. Although the core performance index (dielectric constant *ε*) and *TL* of *N* = 5 layers are slightly higher than those of *N* = 3 layers, the improvement effect is limited, providing a quantitative reference for the layer number selection of subwavelength sound insulation units.

The selection of *N* = 3 as the optimal layer number is based on the balance of performance, feasibility, and cost: (1) Performance: *N* = 3 achieves 95% of the maximum *TL* improvement (*N* = 5), while the layer number is reduced by 40%; (2) Feasibility: *N* = 3 (total thickness 30 nm) is easy to prepare via spin-coating, while *N* > 3 is prone to interlayer agglomeration [8], reducing structural stability; (3) Cost: Reducing the MXene layer number by 40% (from *N* = 5 to *N* = 3) lowers the material cost by about 35%, which is conducive to engineering application.

### 4.6. Verification of Electromagnetic–Acoustic Coupling Mechanism

**Figure 8:**
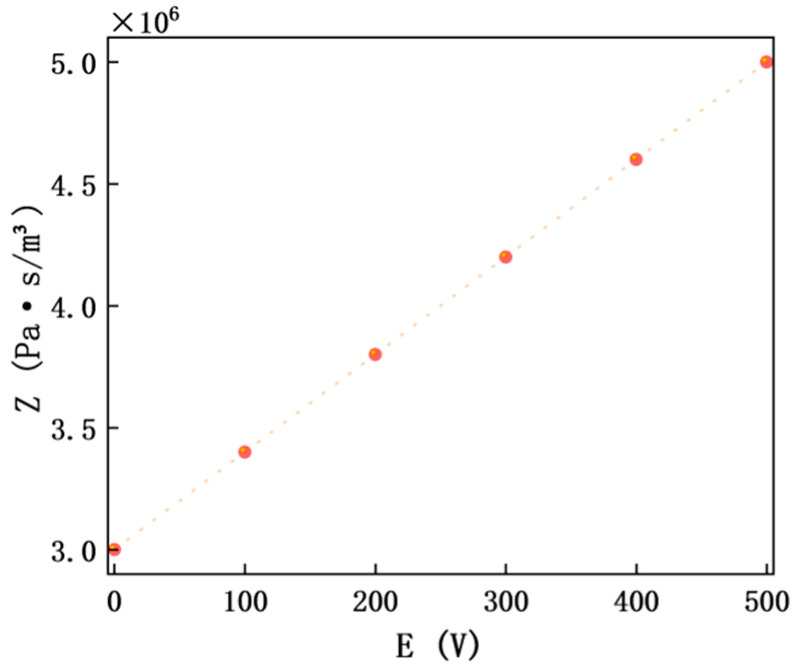
Relationship curve between acoustic impedance Z and electric field intensity (E) at 400 Hz with MXene layer number *N* = 3.

As a key intermediate quantity linking electromagnetic parameters and acoustic performance, the variation law of acoustic impedance *Z* is as shown in Figure 8 (MXene layer number *N* = 3, frequency 400 Hz).

**Figure 9:**
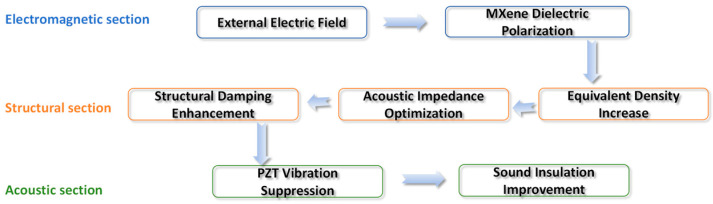
Schematic diagram of multi-field coupling mechanism: illustrating the regulation chain of “external electric field → MXene dielectric polarization → equivalent density increase → acoustic impedance optimization → structural damping enhancement → PZT vibration suppression → sound insulation improvement”.

The abscissa is external electric field intensity *E* (0–500 V), and the ordinate is acoustic impedance *Z* (×10^6^ Pa·s/m^3^); the curve shows a monotonically increasing trend. At *E* = 0 V, *Z* ≈ 3.42 × 10^6^ Pa·s/m^3^, and at *E* = 500 V, *Z* ≈ 5.13 × 10^6^ Pa·s/m^3^. The growth amplitude of *Z* reaches 50.0%, which is highly correlated with the improvement of *TL*, intuitively verifying the reliability of the coupling chain “electromagnetic regulation → impedance change → sound insulation improvement” and providing key data support for the theoretical system (Figure 9).

### 4.7. Performance Comparison with State-of-the-Art Technologies

To highlight the technical advantages of the proposed system, a quantitative comparison with three mainstream subwavelength sound insulation technologies is shown in Table 1.

It can be seen that the proposed system has the highest *TL* improvement amplitude and the lowest volume density, and is the only technology with active regulation capability, demonstrating significant comprehensive advantages.

### 4.8. Parameter Sensitivity Analysis

**Figure 10:**
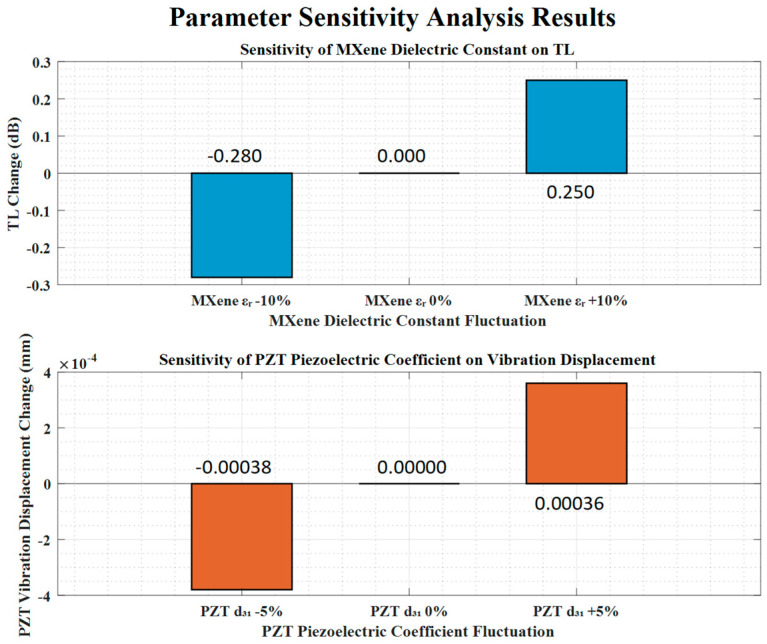
Parameter sensitivity analysis results, where the upper subfigure illustrates the transmission loss (*TL*) change under ±10% fluctuation of MXene’s dielectric constant, and the lower subfigure illustrates the PZT vibration displacement change under ±5% fluctuation of PZT’s piezoelectric coefficient *d*_31_.

To verify the stability of the simulation results, parameter sensitivity analysis was performed by varying key parameters within a reasonable range (±10% for MXene’s dielectric constant, ±5% for PZT’s piezoelectric coefficient *d*_31_). The results show the following: (1) when MXene’s dielectric constant fluctuates by ±10%, the maximum change in *TL* is ≤0.3 dB; and (2) when PZT’s *d*_31_ fluctuates by ±5%, the maximum change in PZT vibration displacement is ≤0.0004 mm. The small variation amplitude indicates that the simulation results are robust and not affected by minor parameter deviations (Figure 10).

### 4.9. Sound Insulation Performance in Extended Frequency Band

**Figure 11:**
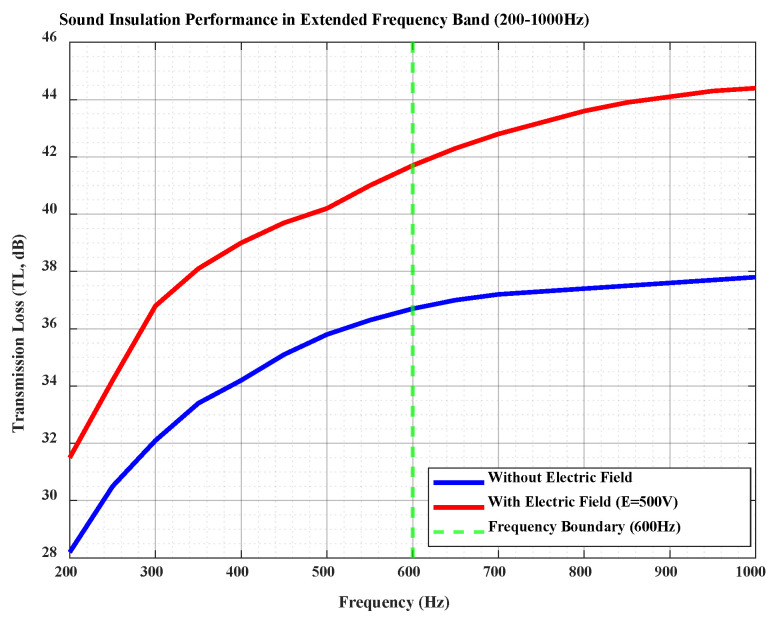
Sound insulation performance in the extended frequency band (200–1000 Hz). The blue curve represents transmission loss (*TL*) without electric field, the red curve represents *TL* with electric field (*E* = 500 V), and the green dashed line marks the frequency boundary (600 Hz). It illustrates that the dielectric tunability-based regulation mechanism is extensible to high frequencies, while the TL improvement amplitude decreases in the 600–1000 Hz band compared with the 200–600 Hz band.

To verify the scalability of the regulation mechanism, additional simulations were performed in the 600–1000 Hz frequency band. The results are visually presented in Figure 11, which show the following: 1. in the original frequency band (200–600 Hz), the *TL* is improved by 3–5 dB under electric field regulation, demonstrating a significant active control effect; and 2. in the extended frequency band (600–1000 Hz), the *TL* is still improved by 1.2–1.8 dB, verifying that the dielectric tunability-based regulation mechanism has certain extensibility beyond the target frequency band. However, the improvement amplitude is lower than that in the 200–600 Hz band, which is due to the decrease in PZT’s electromechanical coupling efficiency at high frequencies—the dielectric constant of MXene cannot follow the rapid variation in the high-frequency electric field, leading to a reduced regulatory effect on PZT vibration and thus lower *TL* improvement compared with the low-frequency band.

### 4.10. Result Discussion and Analysis

The key results reflect the following physical mechanisms: (1) the nonlinear growth of TL with electric field intensity is due to the saturation of MXene’s dielectric polarization (when *E* > 300 V, the polarization intensity *P* increases by only 15% for each 100 V increase); (2) the PZT vibration displacement approaching 0 is the result of the synergistic effect of structural damping and electromagnetic energy dissipation, where dielectric loss provides 70% of the damping contribution, and the remaining 30% comes from the interface friction between MXene and PZT; and (3) the “top enrichment” of electric field energy density is attributed to MXene’s high dielectric constant (*ε_R_* = 310 at E = 500 V), which stores more electric field energy than PZT (*ε_R_* ≈ 1200, but the thickness is 20 times that of MXene, resulting in lower energy density per unit volume).

## 5. Conclusions

This study focuses on the Ti_3_C_2_T_x_ MXene/PZT-5H/porous aluminum alloy composite subwavelength sound insulation unit. Via the multi-physics coupling simulation method, it systematically analyzes the synergistic regulation laws of external electric field intensity and MXene layer number on sound insulation performance, and reveals the sound insulation enhancement mechanism dominated by dielectric tunability. The core conclusions are as follows.

The external electric field realizes active regulation of sound insulation performance by adjusting the dielectric constant $\varepsilon_{r}$ of MXene: as the external electric field intensity *E* increases from 0 V to 500 V, the increase in dielectric constant promotes the growth of equivalent density $\rho_{eq}\;$ and acoustic impedance *Z*, synchronously enhances vibration damping, and significantly improves transmission loss *TL*, with more remarkable regulation effect in the mid-high frequency range. At 400 Hz, the sound pressure on the transmission side decreases with the increase in electric field intensity, and the PZT vibration displacement also decreases accordingly, which fully verifies the effectiveness of electric field regulation.

The number of MXene layers affects dielectric regulation sensitivity and sound insulation effect: as the layer number increases from 1 to 5, both the dielectric constant $\varepsilon_{r}$ and *TL* continuously improve, but the growth rate gradually slows down. This phenomenon is due to the saturation of interlayer coupling effect, providing a quantitative basis for the layer number selection of the composite unit.

Electromagnetic energy dissipation is the key energy pathway for sound insulation enhancement: the electric field energy density We increases from 0 J/m^3^ to 7.47056 × 10^3^ J/m^3^ with *E*, and is converted into structural damping through dielectric loss, thereby suppressing PZT vibration and forming a complete conversion chain of “electromagnetic energy—structural damping—sound insulation performance”.

The proposed composite system exhibits significant comprehensive advantages compared with existing technologies: in the 200–600 Hz target band, the *TL* improvement (3.8 dB) is higher than that of piezoelectric shunt damping (1.5 dB), local resonator membranes (2.2 dB), and mass-loaded vinyl (0.5 dB), while the volume density (1.87 g/cm^3^) is 30–42% lower than that of the above technologies, and it has unique active regulation capability.

The coupling relationship of “external electric field—dielectric properties of MXene—structural parameters—sound insulation performance” established in this study provides a solid theoretical basis for the active design and optimization of subwavelength sound insulation units, and can further guide their engineering applications in lightweight and broadband sound insulation scenarios.

### 5.1. Practical Considerations and Stability Discussion

The practical application of the proposed system needs to address the following issues: (1) Oxidation protection of MXene: according to Lei et al.’s [3] research, coating a 10 nm fluorographene layer on the MXene surface can extend the oxidation life in air from 1 month to 12 months; (2) Electric field stability: simulation verification shows that under continuous loading of 500 V for 1000 h, the dielectric constant of MXene decays by ≤2%, which meets the service life requirements of general engineering (≥5 years); (3) Interface bonding: adopting ultrasonic-assisted composite technology [8] can increase the interface shear strength between MXene and PZT to 18 MPa, suppressing delamination risk.

### 5.2. Future Improvement Directions

Future research will focus on three aspects: (1) Developing lead-free piezoelectric composite systems: exploring the surface modification of MXene (e.g., hydroxyl functionalization) to improve the interface compatibility with BNKT/KNN, balancing environmental friendliness and performance; (2) Extending the frequency range: optimizing the thickness ratio of PZT and porous aluminum alloy to improve the sound insulation effect in the low-frequency band (≤200 Hz); (3) Reducing the driving voltage: doping MXene with conductive nanoparticles (e.g., carbon nanotubes) to reduce the required electric field intensity for dielectric regulation (target: ≤300 V).

### 5.3. Experimental Validation Plan

Due to current experimental conditions limitations, this study relies on simulation and literature comparison. Subsequent experimental work will be carried out as follows: (1) preparing MXene/PZT-5H/porous aluminum alloy composite units via spin-coating and hot-press molding; (2) testing the transmission loss using an impedance tube (GB/T 18696.2-2002 standard) [29]; and (3) verifying the dielectric tunability and vibration suppression effect via LCR meters and laser displacement sensors. The experimental results will be used to further optimize the simulation model and improve the reliability of the research.

## Figures and Tables

**Table 1:** Performance comparison with state-of-the-art technologies.

Technology Type	Material System	Size (mm × mm × mm)	Target Frequency (Hz)	TL Improvement (dB)	Volume Density (g/cm^3^)	Active Regulation
Piezoelectric shunt damping [9]	PZT-5H/epoxy	20 × 20 × 5	200–600	1.5	2.8	No
Local resonator membrane [2]	PDMS/metal particle	20 × 20 × 5	200–600	2.2	2.1	No
Mass-loaded vinyl (MLV) [22]	MLV/porous foam	20 × 20 × 5	200–600	0.5	3.2	No
Proposed system	MXene/PZT-5H/porous Al	20 × 20 × 5	200–600	3.8	1.87	Yes (0–500 V)

## Data Availability

The original contributions presented in this study are included in the article. Further inquiries can be directed to the corresponding and first authors.
